# Biomechanical Modeling of Prosthetic Mesh and Human Tissue Surrogate Interaction

**DOI:** 10.3390/biomimetics3030027

**Published:** 2018-09-18

**Authors:** Arnab Chanda, Tysum Ruchti, Weston Upchurch

**Affiliations:** 1 Department of Bioengineering, University of Pittsburgh, Pittsburgh, PA 15213, USA; 2 Department of Aerospace Engineering and Mechanics, University of Alabama, Tuscaloosa, AL 35401, USA; 3 Department of Mechanical Engineering, Brigham Young University, Provo, UT 84602, USA; tysumruchti@gmail.com; 4 Department of Surgery, University of Minnesota, Minneapolis, MN 55455, USA; wupchurc@umn.edu

**Keywords:** prosthetic mesh, hernia, pelvic organ prolapse, Prolene^®^, finite element

## Abstract

Surgical repair of hernia and prolapse with prosthetic meshes are well-known to cause pain, infection, hernia recurrence, and mesh contraction and failures. In literature, mesh failure mechanics have been studied with uniaxial, biaxial, and cyclic load testing of dry and wet meshes. Also, extensive experimental studies have been conducted on surrogates, such as non-human primates and rodents, to understand the effect of mesh stiffness, pore size, and knitting patterns on mesh biocompatibility. However, the mechanical properties of such animal tissue surrogates are widely different from human tissues. Therefore, to date, mechanics of the interaction between mesh and human tissues is poorly understood. This work addresses this gap in literature by experimentally and computationally modeling the biomechanical behavior of mesh, sutured to human tissue phantom under tension. A commercially available mesh (Prolene^®^) was sutured to vaginal tissue phantom material and tested at different uniaxial strains and strain rates. Global and local stresses at the tissue phantom, suture, and mesh were analyzed. The results of this study provide important insights into the mechanics of prosthetic mesh failure and will be indispensable for better mesh design in the future.

## 1. Introduction

**Figure 1:**
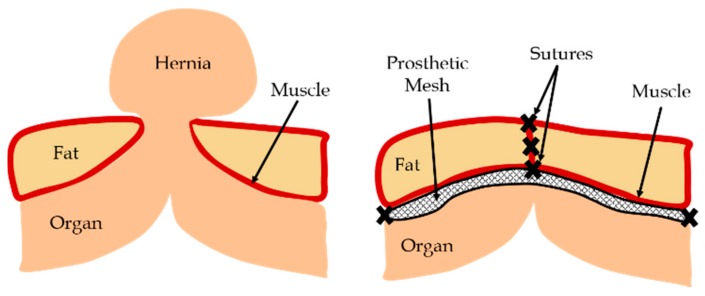
Schematic of hernia and hernia repair using a prosthetic mesh.

Human organs are supported by muscles and various connective tissues. In the event of an injury or deformity which causes tissue weakening or damage, the supporting structures of the organ may fail. A common occurrence due to such events is hernia, where an organ pushes through a tissue or muscle which usually holds it in place (Figure 1). The most common type of hernia is inguinal hernia, where the intestine pushes through the inguinal canal [1]. Other types include incisional, femoral, and hiatal hernias [2]. One example of a displaced organ in females is pelvic organ prolapse (POP), where one or more of the pelvic organs push into the vaginal canal [3]. These gaps can be closed by using prosthetic meshes, which provide flexible support while dispersing the tension from the hernia site. Recently, with advancements in medical diagnosis, several complications associated with surgical meshes have been identified. Some of the major problems reported are mesh contraction and failure, causing tissue straining, pain, infections, and hernia reccurrence. Other common issues with mesh includes mesh erosion, where the surgical mesh wears through nearby soft tissues, and organ perforation, where the mesh cuts through a hollow organ such as the bladder or intestine [4].

Complications arising from prosthetic meshes have led to a significant amount of research into the testing and improvement of commercially available meshes [5]. Besides uniaxial, biaxial, and cyclic load testing on dry and wet meshes [6,7,8], extensive experimental studies have been conducted on animal models [9] to understand the effect of mesh stiffness [10], pore size [11], and knitting patterns [5] on mesh biocompatibility. However, to date, very few studies have investigated the mechanics of meshes implanted within the human body, where the tissue properties are widely different from animal models. Also, no study so far has modeled the mechanical interaction of prosthetic mesh and human tissue. As the meshes do not operate independently and always interact with tissues under tension, a comprehensive understanding of mesh–tissue interactions is warranted [12].

In this work, a commercially available prosthetic mesh was sutured with a vaginal tissue phantom material and tested experimentally to understand the mechanics of the mesh–tissue interaction. The geometry and material property of this mesh and tissue phantom were also recreated within a finite element (FE) model, where they were numerically sutured and tested under tension to characterize the biomechanical contribution of different components in mesh–tissue interactions. The effect of varying strains and strain rates on the uniaxial stress–strain relationships of the sutured mesh and tissue were studied. Section 2 discusses the vaginal tissue phantom fabrication, and the experimental and FE modeling frameworks. Section 3 presents the key results, followed by conclusions in Section 4.

## 2. Materials and Methods

The fabrication methodology of the novel vaginal tissue phantom; experimental testing; FE modeling of the mesh, tissue phantom, and sutures; material modeling; and data analysis are presented in the following sections.

### 2.1. Tissue Phantom Fabrication

**Figure 2:**
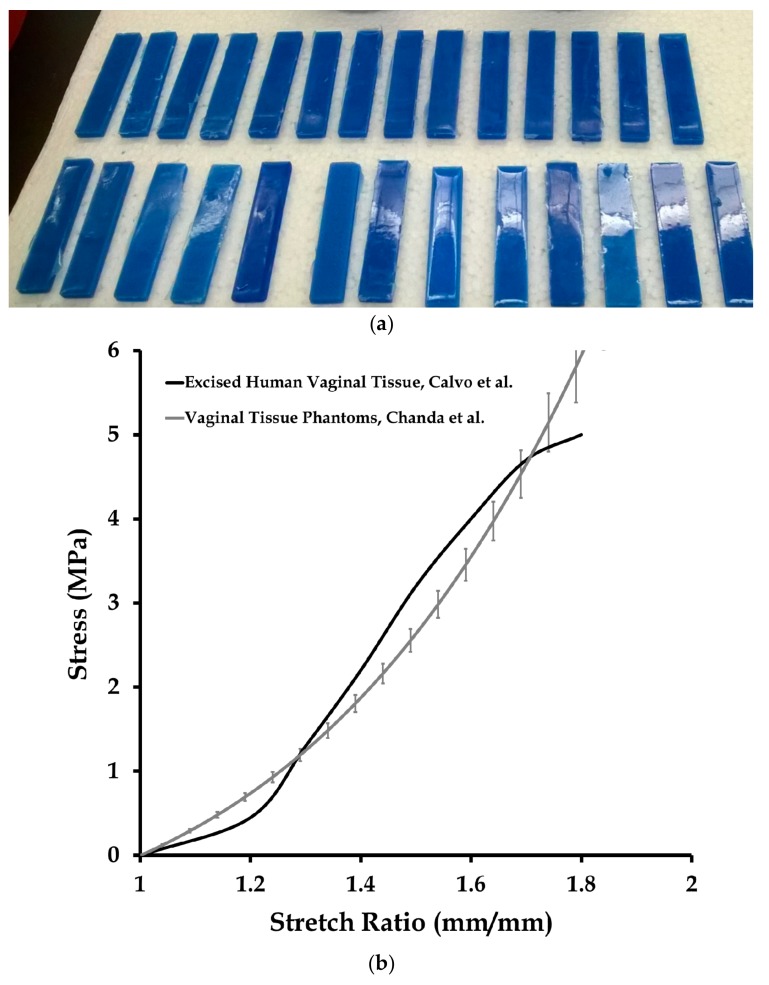
Mechanical testing of vaginal tissue phantoms. (**a**) Vaginal tissue samples. (**b**) Average and standard deviation of stress vs. stretch responses of 27 vaginal tissue phantoms tested by Chanda et al. [13,14,15], compared with average biomechanical properties of excised human vaginal tissue, tested by Calvo et al. [22]. Adapted from [22], Copyright 2009, with permissions from Elsevier. The strain rate for all the experiments was 0.08 mm/s.

Multiple-part elastomers were used to fabricate the vaginal tissue phantoms, which are described in detail by Chanda et al. [13,14,15,16,17,18,19,20,21]. For this study, a two-part elastomer material with shore hardness 10 (Ecoflex^®^ 0010, Smooth-On, Inc., Macungie, PA, USA) and another two-part elastomer with shore hardness 30A (Mold Star 30, Smooth-On, Inc.) were procured and mixed to obtain a four-part mixture. The resulting mixture comprised of 35 wt % of Shore 30A (part A), 35 wt % of Shore 30A (part B), 15 wt % of Shore 10 (part A), and 15 wt % of Shore 10 (part B). Twenty-seven test coupons were casted (Figure 2a) with this mix ratio in a mold, with coupon dimensions of 5 cm × 1 cm × 3 mm. Uniaxial mechanical tests were conducted on the test coupons using a tensile testing machine (MTS Criterion, Model 42, MTS Systems Corporation, Eden Prairie, MN, USA) at a test rate of 0.08 mm/s [13,14,15], the results of which are summarized in Figure 2b. The stress versus stretch results from the tests were compared with the average mechanical property of excised human vaginal tissue samples in literature measured by Calvo et al. at a 0.08 mm/s test rate [22] (Figure 2b).

### 2.2. Experimental Testing

Uniaxial tension tests were performed at different strain rates on the vaginal tissue phantom, prosthetic mesh, and the vaginal tissue phantom sutured to the prosthetic mesh. The prosthetic mesh selected for this study was Prolene^®^ (Ethicon Inc., Somerville, NJ, USA), which has been used previously for hernia repair [23] and transvaginal prolapse repair [24]. The mesh was cut into 30 test coupons with a length and width of 2 ± 0.3 cm and 1 ± 0.1 cm, respectively; 15 out of the 30 mesh samples were tested uniaxially at different strain rates (five sample each at 1, 50, and 500 mm/min, respectively) and compared with results reported in the literature [6]. Also, 12 out the 27 vaginal tissue phantoms were tested at these three strain rates (four sample each at 1, 50, and 500 mm/min, respectively) for further use in the computational model.

The vaginal tissue phantom and prosthetic mesh were connected using sutures to model the initial phase of mesh implantation. Clinically, mesh implantation has been observed to lead to several short-term and long-term problems due to differences in the mechanical properties of the mesh, suture, and native tissue [12]. A typical mesh implantation procedure involves the development of scar tissue, which, over a few weeks, grows through the mesh and subsequently integrates with it to form a new composite made of the native tissue, the mesh implant, and the scar tissues [25]. In this study, the primary focus was to understand the short-term effects of the mesh implantation procedure. Specifically, the mechanical response of the connected mesh, suture, and tissue, and individual components due to different loads (which may vary widely on a subject-specific basis due to factors such as childbirth, physical activity, age, and obesity [3]) was studied. Understanding these initial mechanics of mesh–tissue interaction is the first step towards the investigation of mesh failures, which may provide valuable insights into the formation and mechanical characteristics of the integrated mesh and host tissue. Future studies on the biomechanical modeling of the long-term effects of mesh implantation will be able to provide the entire picture of mesh failure mechanics, and also clarify the contribution of the short-term and long-term effects on mesh failure.

**Figure 3:**
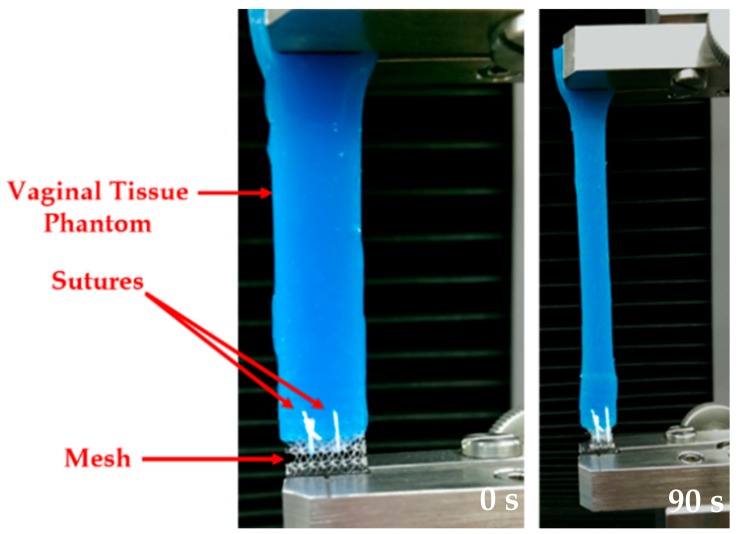
A uniaxial test conducted on a sutured prosthetic mesh and vaginal tissue phantom at 50 mm/min strain rate. Images taken at 0 and 90 s.

In this study, two interrupted sutures were placed (separated by a 2.5 mm distance, and starting at 0.5 mm from the edge of the vaginal tissue phantom) to connect the mesh with the vaginal tissue phantom. It should be mentioned here that the distance between the sutures was chosen arbitrarily to focus mainly on the simulation of sutures, as has been done in previous modeling studies with sutures [16,26]. Clinically, the standard suturing practice has been to maintain a distance between the sutures which is close to the bite distance (distance between the open edge of the tissue or wound, and the point of suture entry or exit) [27]. A size 3-0 polypropylene suture (Ethicon Inc.) and a size 13 sewing needle were chosen for the suturing. Figure 3 shows images at 0 and 90 s during a uniaxial test conducted at a strain rate of 50 mm/min. The experiments were conducted on the sutured prosthetic mesh and tissue at the 1, 50, and 500 mm/min strain rates, selected based on the literature [6].

Stress versus stretch plots were obtained from the uniaxial tensile testing for 90 s on 12 vaginal tissue phantoms, 15 prosthetic mesh samples, and 15 sutured mesh-tissue specimens (groups of five tested at each of the three different strain rates). The nonlinear material properties obtained for the vaginal tissue phantoms and the prosthetic mesh were compared with literature to ensure a high fidelity of test results. The average mechanical properties estimated for the tissue and mesh were used in the finite element model, which is discussed in the following sections.

### 2.3. Finite Element Modeling of Mesh, Tissue, and Sutures

**Figure 4:**
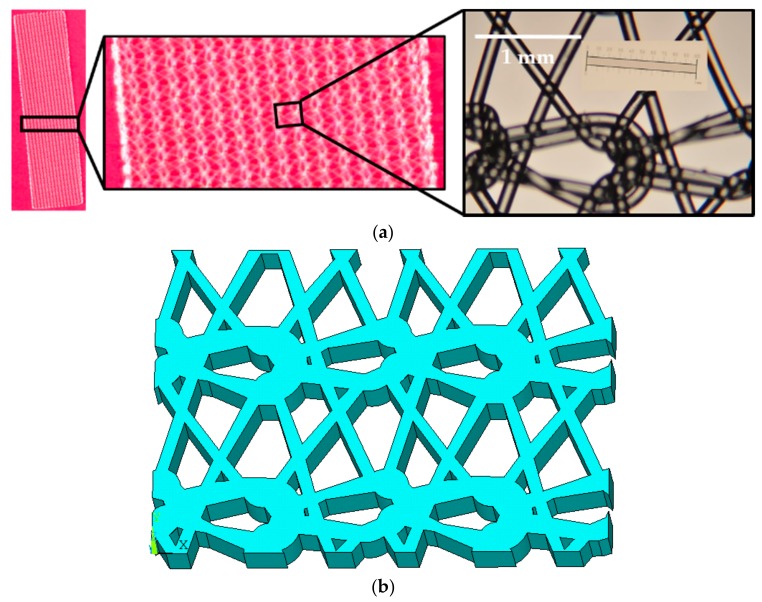
Two steps for prosthetic mesh model development. (**a**) Microscope images of the Prolene^®^ prosthetic mesh showing the repetitive unit. (**b**) Simplified geometric model of the prosthetic mesh.

To develop a geometrical model of the prosthetic mesh, the Prolene^®^ mesh was observed under a regular lab microscope (29AX E250223, Motic Inc., Xiamen, China) operated at a zoom magnification power of 75×. A complex knitting pattern was observed, and several microscopic images were captured and studied to identify the repeating unit (Figure 4a).

**Figure 5:**
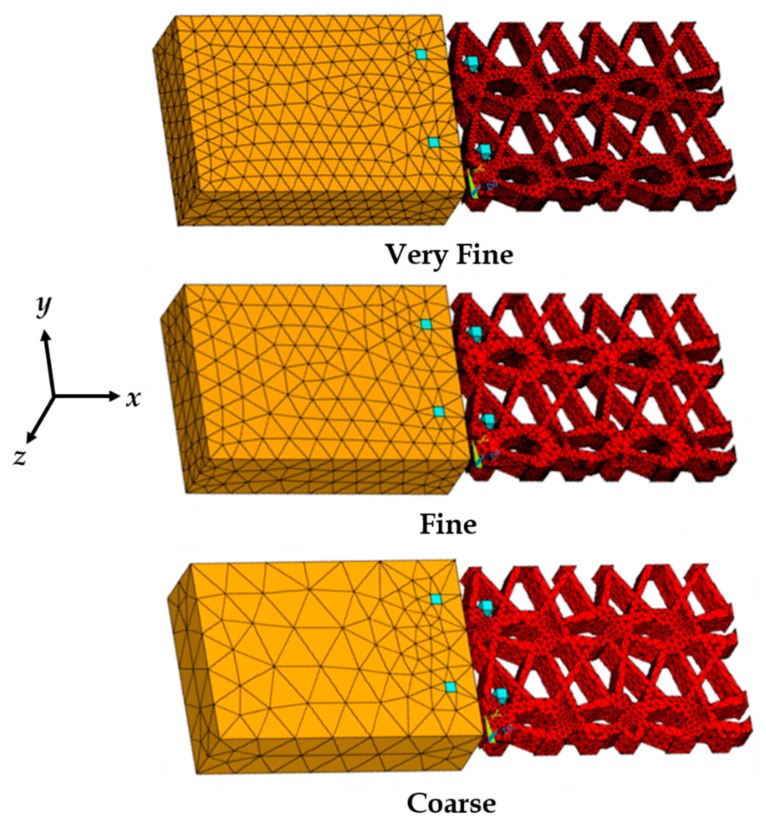
Finite element meshes of the vaginal tissue phantom, prosthetic mesh, and sutures, modeled for the mesh convergence study.

Several points were placed along the prosthetic mesh strands observed in the repetitive unit, and their locations were recorded in a two-dimensional *x*,*y* plane with respect to the origin. The points were plotted in ANSYS APDL 17.1 FE software (Ansys Inc., Cannonsburg, PA, USA) and connected with splines. The lines were converted to areas and the knitted mesh was approximated as a single solid volume (see Figure 4b) with outer dimensions of 5.2 mm × 3.6 mm, and a thickness of 0.6 mm (measured using a vernier caliper). None of the actual prosthetic mesh dimensions were scaled. The vaginal tissue test coupon was modeled as a block with dimensions of 5.2 mm × 3.6 mm × 1.5 mm. The modeling of the sutures (suture sections) are described in detail by Chanda et al. [26,28]. The sutures started at the top of the tissue block with a distance of 0.5 mm from the edge (bite distance), and curved down to exit the tissue block from the side facing the prosthetic mesh (positive *x*-axis) at a depth of 1.0 mm (see Figure 5). The sutures then extended by curving up into the nearest opening of the adjacent prosthetic mesh to hold it against the tissue block. The sutures were modeled with a square cross-section with an edge length of 0.2 mm, approximated to a size 3-0 suture. The reason behind selecting a square cross-section over a round cross-section was for FE mesh optimization, which is discussed by Chanda et al. [26,28,29,30]. Two sutures were modeled, separated by a distance of 2.5 mm, simulating the experimental suture placements. Twenty node Solid 186 elements were used for FE discretization of all the geometrical models. Three FE meshes were generated to conduct a mesh convergence study, including coarse mesh (1292 elements), fine mesh (9704 elements), and very fine mesh (27,528 elements). Figure 5 shows the three FE mesh models, including the vaginal tissue phantom, prosthetic mesh, and two sutures.

The sutures, vaginal tissue phantom, and the prosthetic mesh were connected with two flexible contact pairs (always-bonded type). The first contact pair connected the surface of the sutures embedded within the vaginal tissue block with the tissue. The second contact pair was placed on the surface of the sutures, which was in contact with the mesh at locations of the mesh opening closest to the tissue (see Figure 5). Further details on the characteristics of the contact pair used are described by Chanda et al. [26,28]. With respect to the constraints applied to the FE model, the surface of the tissue block in the *y*,*z* plane facing away from the prosthetic mesh was constrained in all degrees of freedom. Loading was applied by subjecting the free surface of the mesh in the *y*,*z* plane to varying strains and strain rates along the positive *x*-axis, similar to the experiments.

### 2.4. Material Modeling

Soft and flexible materials, such as tissues and meshes, can be characterized using hyperelastic material models, which are able to accurately model the nonlinear stress–strain relationships under tension or compression [16,21,31,32,33,34,35,36,37,38]. Hyperelastic models are based on the strain-energy function (denoted as ψ), which depends on the type of material [39,40]. A hyperelastic model is dependent on either the principal stretches ($\lambda_{1}$, $\lambda_{2}$, and $\lambda_{3}$) or the Cauchy–Green tensor invariants ($I_{1}$, $I_{2}$ and $I_{3}$) [39,41], which are given by $I_{1}=\sum_{i=1}^{3}\lambda_{i}^{2}$, $I_{2}=\sum_{i,j=1}^{3}\lambda_{i}^{2}\lambda_{j}^{2}$ for i≠j and $I_{3}=\prod_{i=1}^{3}\lambda_{i}^{2}$. Some of the well-known hyperelastic models include the Fung, Mooney–Rivlin, Huphrey, Ogden, Veronda–Westmann, and Martin’s models. In this work, the Veronda–Westmann model was used to model the mechanical properties of the vaginal tissue phantom and prosthetic mesh, for their application in the computational model. Specifically, the Veronda–Westmann model was selected because it is based on the experimental testing of tissues such as the skin [39], and has been observed in previous studies to characterize soft phantoms of arterial tissues [20], brain tissues [33], and vaginal tissues [14] with higher accuracy (or degree of correlation) compared to other hyperelastic models. For a uniaxial tension test, Veronda–Westmann’s strain-energy function is given by $\psi =c_{1}[e^{c_{2}(I_{1}-3)}-1]-\frac{c_{1}c_{2}}{2}(I_{2}-3)$, where $c_{1}$ and $c_{2}$ are the hyperelastic curve-fit parameters. The relationship between stress and stretch is derived from $\sigma_{1}=\lambda_{1}\frac{\partial \psi }{\partial \lambda_{1}}-\lambda_{3}\frac{\partial \psi }{\partial \lambda_{3}}$, $\sigma_{2}=\sigma_{3}=0$, and written as $\sigma =2(\lambda^{2}-\frac{1}{\lambda })c_{1}c_{2}(e^{c_{2}(I_{1}-3)}-\frac{1}{2\lambda })$. For accuracy in the quality of hyperelastic curve-fitting, an *R*^2^ correlation estimation was performed for the actual and predicted test plots. The curve-fit parameters for each case were computed to result in an *R*^2^ value in the range of 0.95–1.

The sutures were modeled as linear elastic materials due to their significantly higher stiffness compared to tissues and elastomers (or phantoms). The load-deformation response of sutures is approximately linear, which can be easily characterized using an average Young’s modulus *E* and Poisson’s ratio. In this study, the linear-elastic mechanical properties of sutures were selected based on various synthetic sutures tested by Greenwald et al. [42] and modeled previously by Chanda et al. [21,26]. For the computational study, Young’s modulus of 130 MPa and Poisson’s ratio of 0.23 was assumed for the sutures (Table 1).

### 2.5. Data Analysis

The engineering stress versus stretch results recorded from the mechanical tests were analyzed using Microsoft Excel software (Microsoft Corp., Redmond, WA, USA). The average and standard deviation of stress–stretch were computed and plotted across the number of tests conducted for the vaginal tissue phantoms or prosthetic mesh, for each of the three different strain rates (1, 50, and 500 mm/min). Similar computations were performed for the sutured mesh and vaginal tissue phantom tests at these strain rates. The hyperelastic curve fitting was conducted in three steps. First, the stretch increment values from the experiments were substituted into the Veronda–Westmann's hyperelastic stress–stretch equation (Section 2.4), along with any arbitrary values assigned for the curve-fit coefficients ($c_{1}$ and $c_{2}$). Second, the predicted stress values were subtracted from the experimental stress values for all stretch increments, and squared. Third, these squared values were added for all stretch increments and the minimum sum was computed using the Microsoft Excel curve-fit solver (as the best values of $c_{1}$ and $c_{2}$ were predicted). To determine the accuracy of the estimated curve-fit coefficients, an *R^2^* was computed between the experimental stress values and predicted stress values (with the estimated $c_{1}$ and $c_{2}$) across all stretch measurements. All analyses were conducted with a significance level (*α*) of 0.05. For *R^2^* above a threshold of 0.95, the curve-fit coefficients were considered to be highly accurate. No specific analysis was conducted with the data generated from the computational model (the maximum von Mises stress was computed at different strains and strain rates).

## 3. Results and Discussion

The mechanical testing results of the vaginal tissue phantoms and the prosthetic mesh individually are discussed in this section, followed by the testing of the tissue sutured with the prosthetic mesh. Additionally, the FE mesh convergence study is presented, followed by the results on the effect of different strains and strain rates on the sutured tissue–mesh composite computational model.

### 3.1. Mechanical Testing and Characterization of Vaginal Tissue Phantoms and Prosthetic Mesh

**Figure 6:**
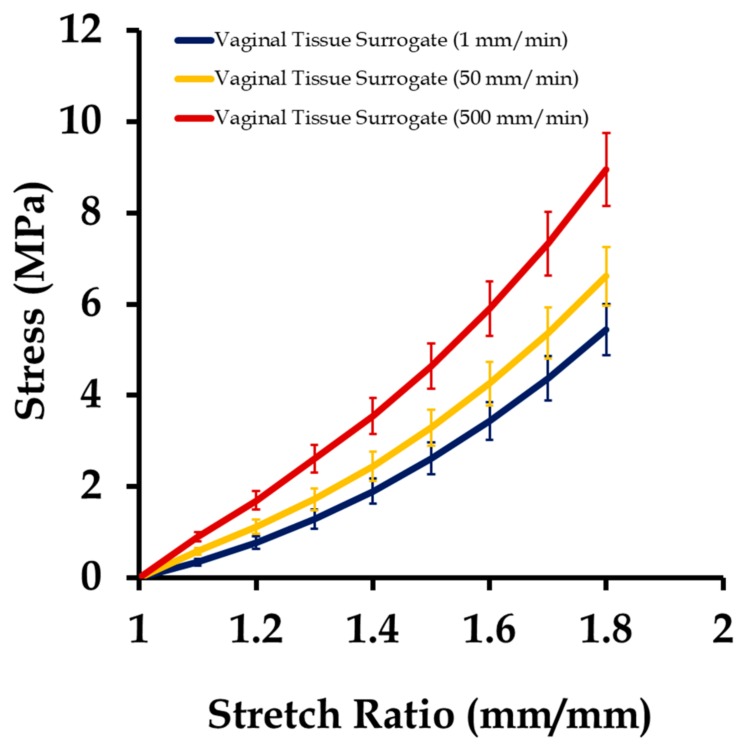
Stress vs. stretch plot of 12 vaginal tissue phantoms tested under uniaxial loading (four samples each at 1, 50, and 500 mm/s). Data are shown as mean ± standard deviation.

The 12 vaginal tissue phantom test coupons were tested uniaxially at three different strain rates (1, 50, and 500 mm/min), as described in Section 2. Figure 6 shows the stress versus stretch results from these tests at different strain rates. Testing at high strain rates resulted in a stiffer mechanical response of the vaginal tissue surrogates, similar to what is observed in the natural tissue and elastomers [3,14]. In a natural tissue, this stiffening effect with increasing strain rate is attributed to the better alignment of the collagen fibers with the loading direction, allowing the tissue to bear higher loads with minimal stretching [43]. The average stress–stretch plot was characterized using the Veronda–Westmann’s hyperelastic model equation introduced in Section 2.4. The values of $c_{1},\;c_{2}$ obtained for different strain rates are presented in Table 1. The average *R*^2^ value obtained from the curve-fitting was quantified to be 0.965. These results were compared with vaginal tissue surrogates tested in [14,44], and were found to be within the range of parameters and correlation values reported previously.

**Figure 7:**
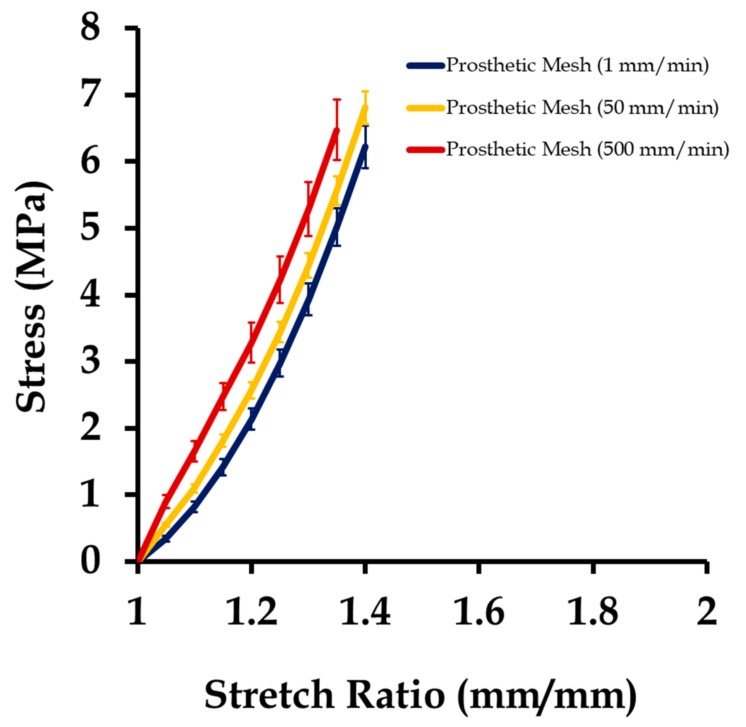
Stress vs. stretch plot of 15 prosthetic mesh samples tested under uniaxial loading (five samples each at 1, 50, and 500 mm/s). Data are shown as mean ± standard deviation.

The results from testing of the prosthetic mesh samples are summarized in Figure 7. The average plots were compared with testing on Prolene^®^ meshes [6], and were found to be within the range of the mechanical properties reported. Overall, the prosthetic mesh mechanical behavior was observed to be stiffer than the vaginal tissue surrogates, and changed minimally with strain rates. All meshes underwent permanent deformation after a stretch of 1.22 ± 0.23 and reached ultimate tensile stress at an average stretch of 1.39 ± 0.11. This observation was different from the case of the vaginal tissue phantom tests, where the permanent deformation was not apparent up to an average stretch value of 2.2. On fitting the average stress–stretch data of prosthetic mesh samples into Veronda–Westmann’s hyperelastic constitutive relationship, the values of the parameters obtained had an average *R*^2^ value of 0.981. Table 1 summarizes the average hyperelastic parameters estimated for the vaginal tissue phantom, prosthetic mesh, and the linear elastic properties adopted for the sutures from the literature for use in computational modeling.

### 3.2. Mechanical Testing of Sutured Prosthetic Mesh–Vaginal Tissue Phantoms

**Figure 8:**
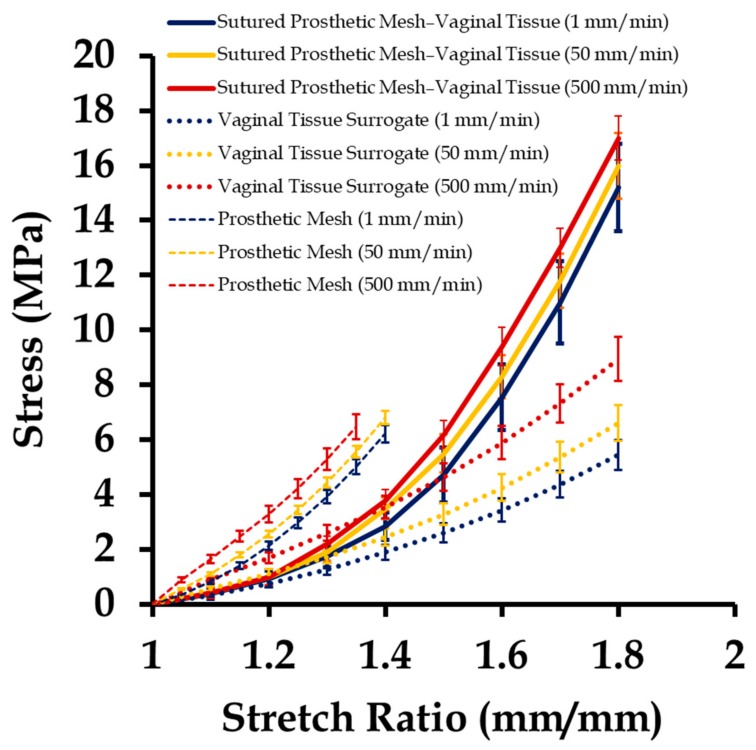
Uniaxial stress vs. stretch plot of 15 sutured prosthetic mesh and vaginal tissue phantom at strain rates of 1, 50, and 500 mm/min (five samples at each strain rate), compared with the stress–stretch responses of individually tested 12 vaginal tissue phantoms (four samples at each strain rate) and 15 prosthetic mesh (five samples at each strain rate). Data are shown as mean ± standard deviation.

Sutured prosthetic mesh and vaginal tissue phantoms were tested at three different strain rates, the results of which are shown in Figure 8, along with the stress–stretch plots of the individually tested vaginal tissue phantoms (Figure 6) and the prosthetic mesh (Figure 7) in the previous section. Several observations were recorded. Firstly, the stress–stretch response of the sutured prosthetic mesh and vaginal tissue phantom composite was similar to the stress–stretch profile of the vaginal tissue surrogate, which was observed up to an approximate stretch value of 1.5. Secondly, permanent deformation in the mesh was observed beyond an overall stretch of 1.5. Thirdly, the stress–stretch plots of the sutured prosthetic mesh and tissue phantom lied in between that of the prosthetic mesh and vaginal tissue phantom tests. Fourthly, the mesh was observed to twist in the clockwise direction about the loading axis (*x*-axis). Fifthly, strain rates varying from 1 to 500 mm/min did not have any significant impact on the stress–stretch plots of the sutured prosthetic mesh and vaginal tissue phantom. This finding may be explained with the non-significant variations observed with the strain rate on the mechanical testing of Prolene^®^ prosthetic mesh [6], which has a possible dominant effect on the overall stress–stretch response compared to the biomechanical variations typically observed in a tissue or tissue phantom with changes in strain rate [33,34,35,36,37,38,39,40,41,42,43,44,45].

### 3.3. Finite Element Mesh Convergence Results

**Figure 9:**
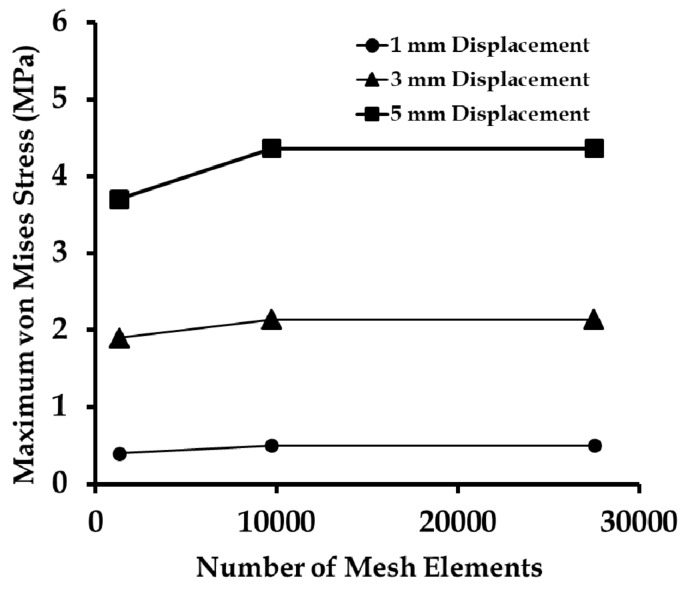
Maximum von Mises stress induced due to three different displacements applied on finite element meshes with a different number of elements, as part of the mesh convergence study.

The three FE meshes introduced in Section 2.3 (Figure 5) were subjected to 1, 3, and 5 mm uniaxial (positive *x*-axis) displacements at the mesh end, representing approximately 10%, 30%, and 50% of strains, respectively. The maximum von Mises stress was quantified for the three different FE meshes with 1292, 9704, and 27,528 elements (Figure 9). It was observed from the study that for a coarse mesh with a total number of elements of less than 9704, the von Mises stress changed with the mesh density up to a maximum of 0.74 MPa for a 5 mm displacement. However, going from 9704 (fine mesh) to 27,528 elements (very fine mesh), the change in von Mises stress for all displacements were negligible. Therefore, the fine mesh with 9704 elements was considered to be computationally accurate and an efficient mesh for further study.

### 3.4. Effect of Varying Strains on the Sutured Prosthetic Mesh–Vaginal Tissue Phantom Finite Element Model

The effect of uniaxial strain (or stretch) on the sutured vaginal tissue phantom and prosthetic mesh model was studied to identify how it affected the induced displacements and stresses on the individual components (i.e., vaginal tissue, suture, and prosthetic mesh). Stretch values were applied in the range of 1–1.5 at a strain rate of 1 mm/min with five load steps to compare with the experiments. The material properties were selected for the mesh and vaginal tissue phantom from the experiments at this strain rate (Section 2.4).

**Figure 10:**
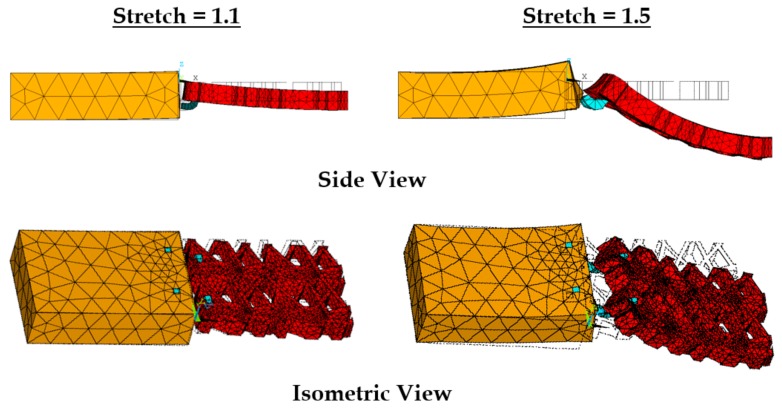
Displacements observed at the vaginal tissue phantom, prosthetic mesh, and sutures, at stretch values of 1.1 and 1.5, respectively.

**Figure 11:**
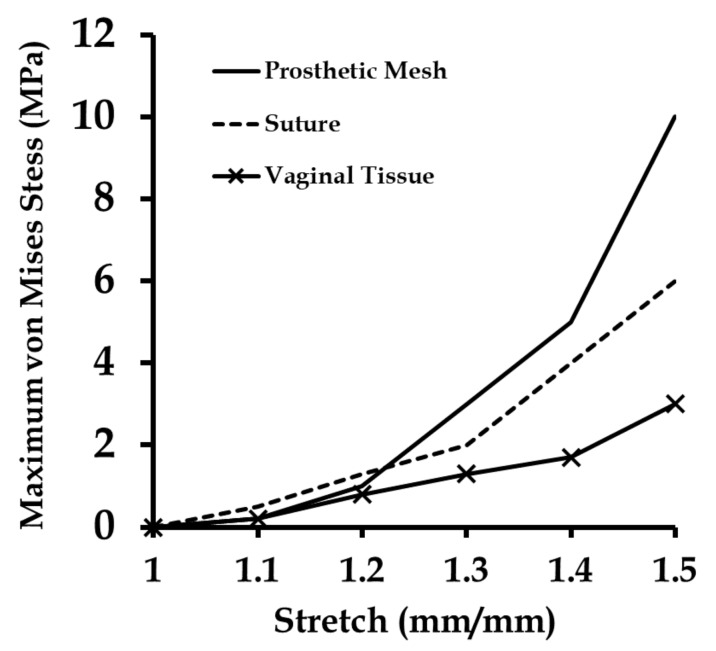
Maximum stresses induced at the prosthetic mesh, suture, and vaginal tissue phantom computational models as they were stretched together in uniaxial tension in five load steps (at a strain rate of 1 mm/min).

With stretching, the prosthetic mesh was found to displace significantly in the *z* direction along with the *x*-displacement (see Figure 10). Also, a clockwise twist was observed about the *x*-axis, which was also observed in experiments. The maximum stress on the prosthetic mesh was found to be concentrated at the location of its attachment with the sutures. Also, this maximum stress value of 1.5 (10.2 MPa) was significantly higher than the average stress in the tissue phantom–suture–mesh composite system recorded in the experiments (6.1 MPa). Other significant stress concentration zones included the two curved strand rows in the prosthetic mesh. Figure 11 shows the maximum stresses observed at the vaginal tissue, sutures, and prosthetic mesh due to varying strains. The vaginal tissue was found to be the least stressed component. The sutures were initially the most stressed component compared to the mesh. However, beyond a stretch value of approximately 1.2, the stress in the prosthetic mesh was observed to increase above the stress in the sutures. These findings suggest that, initially, the sutures support most of the tensile load, and prosthetic mesh loading starts around a stretch of 1.2, following which the mesh is the main load-bearing component. This observation is consistent with the clinical finding that the prosthetic mesh starts to curl and deform permanently with increased loading, without significantly stressing the vaginal tissue.

### 3.5. Effect of Varying Strain Rates on Sutured Prosthetic Mesh–Vaginal Tissue Phantom Finit Element Model

**Figure 12:**
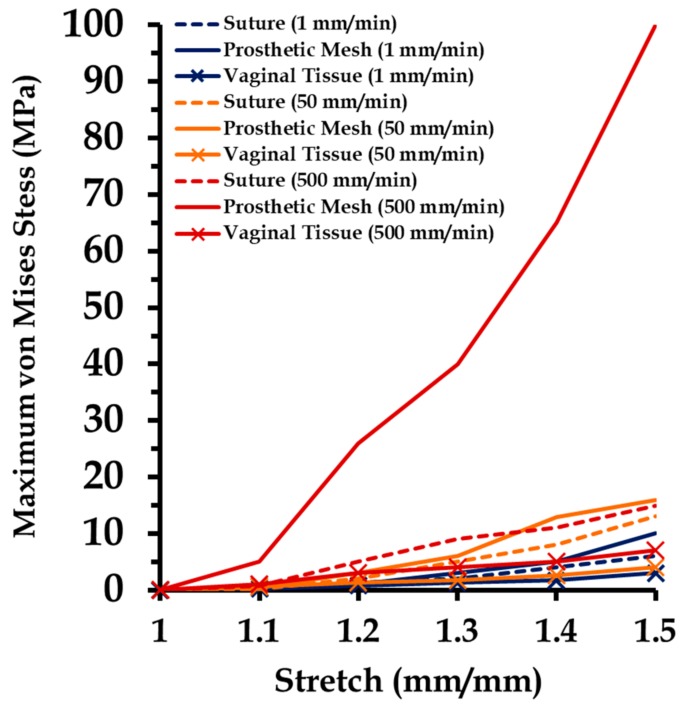
Maximum stresses at the prosthetic mesh, suture, and vaginal tissue panthom computational models stretched at different strain rates (1, 50, and 500 mm/min) in five load steps.

Varying strain rates were applied on the sutured tissue and prosthetic mesh FE model to investigate induced stress distributions on the individual components. Material properties were adopted for the mesh and vaginal tissue phantom from the experiments at different strain rates (Section 2.4). Figure 12 shows a summary of these results. The maximum von Mises stress at the vaginal tissue was not found to change significantly going from strain rates of 1 mm/min (3.3 MPa) to 500 mm/min (7.1 MPa). This finding indicates that the amount of loading on the vaginal tissue was minimal, because stresses in tissues are generally known to change with loading at high strain rates [19,33] (as also observed in the experiments). Comparatively, higher maximum stresses were recorded at the sutures in the range of 6.3–15.5 MPa compared to vaginal tissue surrogates, with increasing strain rates. The stresses studied at the prosthetic mesh were different than expected in the experiments. For high strain rates (50–500 mm/min), the prosthetic mesh was quantified to be the major load-bearing component throughout. A significant increase in the maximum stress was observed at a strain rate of 500 mm/min, going up to 101.3 MPa at a stretch of 1.5.

**Figure 13:**
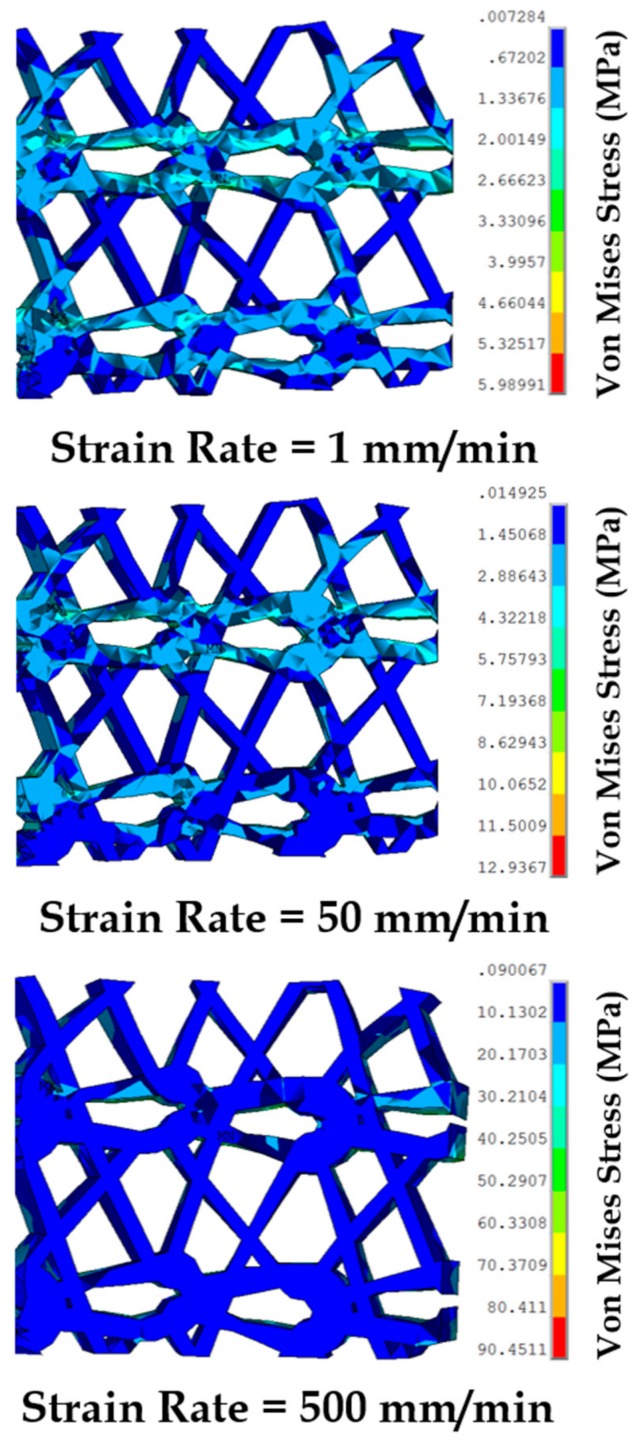
Stress distribution in the prosthetic mesh sutured to the vaginal tissue phantom, observed at a stretch of 1.4 during uniaxial tension tests conducted at varying strain rates.

**Figure 14:**
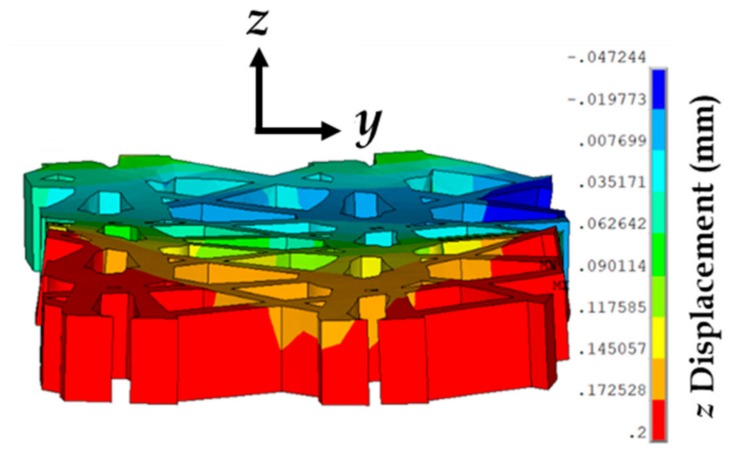
Representation of the prosthetic mesh showing a slight curling due to large and small *z*-displacements at the edges and mid-plane, respectively, for a tissue phantom–suture–mesh composite stretch of 1.4 and strain rate of 1 mm/min.

To capture the stress distributions in the highly loaded prosthetic mesh at different strain rates, snapshots of the von Mises stress test were obtained at a stretch of 1.4 (Figure 13). This specific stretch value was selected because the prosthetic mesh was observed to undergo permanent deformation beyond this value in these experiments. At 1 mm/min, the stress distribution was observed to be localized at the location of suture attachments and the two sets of curved strands in the prosthetic mesh along the *x*-axis. Minimal stress was observed in the linear strands connecting the two sets of curve strands. Specifically neglecting the highest stress observed at the location of suture attachment, the maximum stress (5.9 MPa) was localized close to certain edges in the top curved strand set (see Figure 13). One explanation for this stress localization is the better alignment of the top curved strand set with the uniaxial loading axis observed in the FE model compared to the bottom curved strand set, after stretching and slight curling (Figure 14) in the prosthetic mesh. With an increase in strain rate from 1 to 50 mm/min, the stress distribution migrated significantly from the two curved strand sets to mainly the top curved strand set. The maximum stress value (12.9 MPa) was reported at the same edges, which was observed at 1 mm/min strain rate. At a strain rate of 500 mm/min, the stress distribution completely moved to the top curved strand set with a significantly high maximum stress distribution of 90.4 MPa at the edges identified earlier. Such high-stress concentration zones may be possible sites of mesh failures. Having a closer look at the specific prosthetic mesh edges where the maximum stress occurred, we found that it was actually just one edge within the repetitive unit which was used to construct the numerical prosthetic mesh model (see Figure 4). Thus, it may be possible that the structural repetitive unit of a prosthetic mesh influences the stress distribution within the loaded sutured mesh and tissue. Experimental investigation of this finding with imaging techniques such as the digital image correlation will be a subject of future studies.

## 4. Conclusions

Here, we studied the complex interaction between a sutured prosthetic mesh and soft tissue under tensile loading. A novel experimental framework was developed to conduct uniaxial tensile tests on a commercially available prosthetic mesh (Prolene^®^) sutured with a vaginal tissue phantom. Average stress versus stretch responses were recorded at varying strains and strain rates for the vaginal tissue phantom and prosthetic mesh individually, and for the sutured vaginal tissue phantom and prosthetic mesh composite. The results indicated a high and low stress–stretch response of the prosthetic mesh and vaginal tissue phantom, respectively, with the stress–stretch of the sutured composite lying in between the two. Also, the sutured composite material properties were observed to be minimally affected by varying strain rates in these experiments. A biofidelic computational model was developed, where the prosthetic mesh (modeled using microscopy) was numerically sutured with a vaginal tissue phantom to simulate the experiments and study induced stresses in the individual components. With increasing strains, the highest loading was found to occur at the location of the sutures, followed by the prosthetic mesh strands. Specifically neglecting the highest stresses observed at the suture locations, high strain rates were found to significantly increase and localize stresses at certain mesh strands. A closer look at the location of the highly stressed prosthetic mesh strands revealed the possibility of some relationship with the mesh repetitive unit structure. Further experimental testing with different tissues, prosthetic meshes, and sutures will be able to further explain these findings and provide important guidelines for prosthetic mesh design and testing in the future.

## Figures and Tables

**Table 1:** Material properties used for the computational model.

Veronda–Westmann’s Hyperelastic Model				Linear Elastic Model	
Material	Strain Rate (mm/min)	$c_{1}\;(\mathrm{MPa})$	$c_{2}\;(\mathrm{MPa})$	*E* (MPa)	Poisson’s Ratio
Vaginal Tissue Surrogate	1	5.6	0.18	-	-
	50	6.1	0.21	-	-
	500	7.4	0.25	-	-
Prosthetic Mesh	1	10.1	0.30	-	-
	50	10.5	0.31	-	-
	500	11.1	0.37	-	-
Suture	-	-	-	130	0.23
